# “I want to be there for my children”: fatherhood, diabetes and temporality among Peruvian men

**DOI:** 10.3389/fcdhc.2023.1207028

**Published:** 2023-10-12

**Authors:** M. Amalia Pesantes, Isabella Ferrazza, J. Jaime Miranda

**Affiliations:** ^1^ Department of Anthropology and Archaeology, Dickinson College, Carlisle, PA, United States; ^2^ Universidad Peruana Cayetano Heredia, CRONICAS Centro de Excelencia en Enfermedades Crónicas, Lima, Peru; ^3^ Facultad de Medicina, Universidad Peruana Cayetano Heredia, Lima, Peru

**Keywords:** diabetes management, masculinity, fatherhood, diabetes, Latin America

## Abstract

**Introduction:**

Living with a chronic condition is a challenging experience, as it can disrupt your capacity to function and fulfill social roles such as being a father. Fatherhood constitutes an important component of masculinity that has not received significant attention in studies aimed at understanding the role of gender norms in health-related behaviors. Fatherhood refers to the set of social expectations placed on men to provide, protect, and care for those considered his children. Our paper aims to show the importance of understanding men’s perspectives around fatherhood and its relevance for staying healthy.

**Methods:**

In-depth semi-structured interviews with men living with Type 2 diabetes in Peru to explore their experiences with diabetes management.

**Results:**

Eighteen Peruvian men, diagnosed with Type 2 diabetes for at least one year and with ages between 27 and 59 years old were interviewed. They had an average of three children each and were all insured under the national insurance plan aimed at low-income groups in Peru. Their accounts described their concern of not being able to fulfill their roles as fathers as a result of their condition. They mentioned the importance of being physically and emotionally present in the lives of their children, taking care of them, and being an example. These concerns varied depending on the age of their children: those with younger children were more preoccupied with ensuring they stayed healthy. Temporality provides a relevant analytical approach to understand the interplay of fatherhood and motivation for diabetes management.

**Discussion:**

Our study advances the research around the intersection between health and gender norms and argues that a more nuanced understanding of the construction of masculinity and the relevance of fatherhood in the lives of men could be useful to design and identify better health promotion strategies tailored to men with diabetes.

## 1 Introduction

Gender norms play an important role in health-related behaviors and have been identified as a key factor to understanding differential vulnerability to illness, access to preventive and curative measures, and quality of care received at home and at the local health facilities. Studies in high income countries have found that masculine gender norms often have a negative impact on the health status of individuals, as men are more hesitant or reluctant than women to seek medical care for various health problems (1–3) and manage a chronic condition (4, 5). Yet, there is a limited number of studies that explore the way masculinity, and the social roles attached to masculinity, plays in health-related behavior among men from low socioeconomic groups in low- and middle-income countries (LMIC).

Individuals fulfill a variety of social roles; such social roles are shaped by cultural norms and expectations closely tied with the individual’s gender and age. One social role that has been identified as important in the construction of masculinity is fatherhood. Fatherhood refers to the set of culturally defined behaviors, emotions, and relations, as well as the duties, rights, and responsibilities of a male towards a child recognized as his own, regardless of whether a biological connection exists (6, 7). In this sense, fatherhood is not a biological fact but a social role and as such, one that varies across cultures, changes overtime in the life of a man, and one that can be impacted by a chronic condition. Several authors have shown that a chronic condition disrupts a person’s biographical trajectory and affects their sense of self, often in negative ways (8–12). However, we are interested in understanding how the desire to continue to fulfill a social role can be a source of motivation to remain healthy; a topic that for the case of chronic conditions such as diabetes is largely understudied, particularly in the Latin American setting. In this article, we examine the significance that men’s expectations around their roles as fathers play in their attitudes and actions towards diabetes management,

The management of Type 2 diabetes, hereinafter diabetes, requires introducing changes in a person’s everyday life. Individuals have to modify their diet, increase the level of physical activity, be consistent with medication intake, and attend follow-up appointments. Diabetes management is crucial to prevent negative health outcomes associated with sustained high glucose levels, such as cardiovascular disease, kidney disease, neuropathy, blindness, and lower-extremity amputation (13). Introducing such behavioral changes has been recognized as challenging, and that requires cultural and gender adaptations for them to be successful. Our study points to the relevance of fatherhood as a motivating factor for diabetes management among Peruvian men, and highlights the role that both biological and social factors play in the meaning and perception of illness experience (14, 15).

In the Latin American context, fatherhood has been identified as an important aspect of the construction of adult masculinity. Studies in this region have highlighted that becoming a father is a rite of passage to adulthood (16, 17). Fatherhood has also been identified not only as a milestone but as being of high importance in their gender identity (18, 19). The few studies around men’s roles and participation in the lives of children in Latin America point to the fact that despite men being less involved in childcare, they do make decisions about the use of household income for their children’s well-being, including education and healthcare (20). Ethnographic research has found evidence that contradicts certain stereotypes about Latin American men, such as “machos” who have several children (as a sign of sexual potency) and are then not responsible for them. A study among Mexican men showed that being worthy of your children’s trust and being committed to fulfilling your obligations as a father plays a central role in the ideal of manhood (21). Other studies in Latin America show that fatherhood occupies an important space in men’s life projects, but they have not looked at the intersection with health concerns (16, 21–24). Studies in Western contexts have also noted that past prevalent models of fatherhood (e.g. the father as the absent breadwinner) have changed (25), and that fathers are progressively not only expected to fulfill the traditional roles of procreation and provision, but also of physical and emotional care and nurturance (26). Based on the accounts of Peruvian men living with Type 2 diabetes, we present and discuss the centrality of their role as fathers in their motivation to follow diabetes management recommendations. Our study aims to contribute to the literature around the intersection of masculinity and health-related behaviors. Such literature often highlights the negative impact of male gender norms in health attitudes and behaviors; however, as our study suggests, male gender norms could also provide opportunities for health promotion.

## 2 Materials and methods

This qualitative study explored Peruvian men’s experiences living with and managing diabetes. Between June and August of 2022, we conducted in-depth semi-structured interviews with 18 men diagnosed with Type 2 diabetes who used public health facilities and lived in Lima and Pucallpa, two cities in Peru. The rationale behind selecting a major coastal city (Lima) and a smaller Amazonian one (Pucallpa) was because one axis of analysis of the broader research project was the relationship of participants with the healthcare system. We wanted to understand if living in the capital city with a wide variety of healthcare options played a role in the relationship between the patient and the health system. However, in terms of the relationship of fatherhood and disease management that guides the analysis we present in this paper, we did not find any significant distinction between the experiences of men in Lima or Pucallpa, and because of this, we do not present data separated by research site.

Interviews lasted an average of 35 minutes (the shortest one was 21 minutes and the longest one was 70 minutes), were conducted in person, in Spanish, by a medical anthropologist whose mother tongue is Spanish, and in spaces convenient for the participants. Participants received a compensation of 100 soles (~ $30) and received detailed information of the study, expected participation, and risks before being asked to sign a consent form. The interview guide covered several topics: health-seeking behaviors to get a diagnosis, relationship with healthcare providers, challenges to access adequate healthcare, impact of the condition in their work or employment status, efforts to incorporate behavioral changes in their everyday lives, and the role of family in such efforts. We placed emphasis on the challenges of living with diabetes and its implications in the everyday lives of these men. As we analyzed their perspectives on disease management, being a father and caring for their children emerged as a core element of their narratives. However, there were other relevant topics our data analysis identified such as the importance of family involvement in diabetes management and the different ways in which children, spouses and friends provide support o ensure the participant incorporated and maintained the behavioral changes suggested by doctors. This is the topic of another article.

## 3 Data analysis

All interviews were transcribed verbatim. To ensure one of the co-authors with a moderate understanding of Spanish could read the transcripts, they were translated into English using a translation program through Microsoft Word. Clarifications from the Spanish-speaking members of the team were requested when needed. Once translated, MAP and IF spent time familiarizing themselves with the transcripts, reading and re-reading them, and adjusting an initial version of the codebook designed by the first author based on preliminary themes. As the transcripts were discussed, it was necessary to add emerging themes to the codebook after deliberating on their pertinence, necessity, and importance. After the codebook (See **Table 1**) was finalized, the analysis went through three stages using a thematic approach:

**Table 1:** Codebook.

CODES AND SUBCODES	DEFINITION
A. BIOGRAPHICAL INFORMATION [SUBCODES: BASIC DEMOGRAPHIC DATA; FAMILY RESPONSIBILITIES]	Participants’ past family life, past experiences with disease and health services. Current family life (who he is living with, what are different responsibilities at home and to sustain the family, etc.)
B. INTERPRETATION OF CONDITION [SUBCODES: CAUSE FOR CONDITION; HOW THE DISEASE WORKS; INFORMATION PROVIDED BY HEALTHCARE PROFESSIONALS]	Patient’s explanation about why they have the condition (ex: what triggered the disease) Including how patients define/interpret/understand their diagnosis Explanations given to participants by health professionals regarding (a) the causes, (b) consequences of the condition and (c) management practices
C. ENGAGEMENT WITH HEALTHCARE SYSTEM [SUBCODES: WILLINGNESS TO INTERACT WITH BIOMEDICINE; NEGATIVE INTERACTIONS; POSITIVE INTERACTIONS]	Patient’s stories about their decisions to search or not for health care, at the different stages of the condition, with special emphasis on regular check-up appointments and when a complication starts. Includes all mentioned negative/positive experiences at said healthcare facilities, can include any stories with or without medical personnel.
D. TREATMENT [SUBCODES: AVAILABILITY AND ACCESSIBILITY; TREATMENT PLAN; INTERPRETATION OF TREATMENT IN RELATION TO THEIR BODIES]	Availability and accessibility of medications/medical procedures/overall care as well as procurement strategies. Any ideas or personal feelings about what the treatment is doing to their bodies (good or bad), specifically other organs/bodily functions
E. MANAGEMENT OF CHRONIC CONDITION [SUBCODES: PHYSICAL ACTIVITY; DIETING; SEEKING ALTERNATIVE TREATMENTS; IMPACT ON SOCIAL LIFE]	Physical activity, treatment adherence, any mention or discussion of dietary habits, specifically the challenges of following a specific diet and any interpretations of “diabetic-friendly” foods – how does this impact their social life? Any decisions made on the basis of their diabetes, specifically seeking out alternative treatments from non-traditional/traditional medical practices
F. FAMILY/FRIEND INVOLVEMENT [SUBCODES: SUPPORT; HINDERANCE]	Description of the type of support (emotional, instrumental, financial, etc.) support received by participants to manage diabetes.
G. FATHERHOOD	Descriptions of participants role in the family as a father – w*hat are his responsibilities? Is fatherhood a motivator to engage in treatment adherence?*

By participant: We identified relevant quotes and information for each code and created matrices for each participant. When reporting on important themes and selecting quotes, the segments were kept in Spanish, as not to misinterpret or change the meaning of the participant’s narratives. One researcher coded all the interviews from Lima and another one all the interviews from Pucallpa, with weekly meetings to ensure consistency.

By code: Individual word documents per code were prepared and then discussed in weekly meetings to summarize the information and take notes to connect the data with the literature and begin the interpretation. In this process, fatherhood was identified as an important issue, therefore, we decided to do a third round of coding for Fatherhood.

Using MaxQDA: IF conducted a last round of coding with specific focus on themes, notions, and mentions of fatherhood using the qualitative coding software MAXQDA 2020. Codes were inserted into the system and used to do a final round of systematic analysis on all 18 transcripts. The data surrounding fatherhood was retrieved from each document and exported as “coded segments” to be interpreted within the context of the research topic. Fatherhood, at this stage was defined as follows: any mention of the participant’s role in the family as a father, specifically the emotional, physical, and social responsibilities he embodies for his children. As the data was coded, we paid particular attention to how these internalized duties motivate the participant to engage in diabetes management practices, as well as how the responsibilities of a father expand or change as the children grow older.

The relevance of fatherhood in men’s narratives of living with diabetes emerged from the interviews conducted, that of which we discuss at length in the following sections.

## 4 Results

### 4.1 Participants

We interviewed a total of 20 men, out of which 18 were parents. The present manuscript focuses on the accounts of those 18 men and exclude those who did not have children. The age of the participants ranged from 27 to 68 years old, with most of them (12/18) being in the age range of 46-60 as shown in **Table 1**.

**Figure 1:**
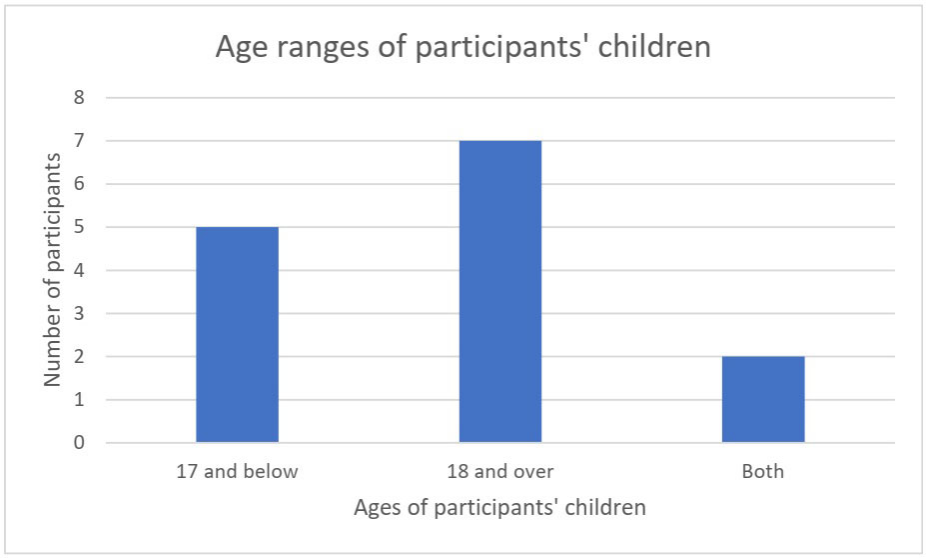
Age ranges of participant’s children.

The average number of children was three (S.D. 1.86). As shown in **Figure 1**, out of the 14 participants who provided information on the ages of their children and/or grandchildren, five had only children between the ages of 0 to 17 years, while seven had children older than 18 years (the age at which you are legally recognized as an adult in Peru). Two participants had both young (under 18) and adult children, eight participants had grandchildren, and the average number of grandchildren was two.

The average number of years living with a diabetes diagnosis for all 18 participants was 7.6 years (S.D. 6.74), however, there was a wide diversity in the number of years these participants have been diagnosed, as shown in **Table 2**.

**Table 2:** Participants’ characteristics.

Pseudonym	Age Ranges (yrs)	Years living with Diabetes	Marital Status	Number of Children	Ages of Children (yrs)	Number of Grandchildren (if mentioned)
*Arturo*	25 to 35	2	Married	4	17 and BELOW	
*Ignacio*	36 to 45	11	Married	3	17 and BELOW	
*Jorge*	36 to 45	2	Married	2	17 and BELOW	
*Norberto*	36 to 45	10	Single	1	17 and BELOW	
*Otilio*	36 to 45	3	Married	3	Not Mentioned	
*Esteban*	46 to 50	8	Married	1	17 and BELOW	
*Kevin*	46 to 50	8	Divorced	3	Not Mentioned	
*Dario*	51 to 60	2.5	Widower	2	18 and OVER	1
*Facundo*	51 to 60	8	Married	2	18 and OVER	1
*Giacomo*	51 to 60	2	Married	2	18 and OVER	1
*Helder*	51 to 60	3	Married	5	17 and BELOW and 18 and OVER	
*Leandro*	51 to 60	17	Married	3	18 and OVER	2
*Mauro*	51 to 60	6	Married	9	17 and BELOW and 18 and OVER	
*Paco*	51 to 60	9	Married	2	18 and OVER	
*Samuel*	51 to 60	4	Married	4	18 and OVER	3
*Teofilo*	51 to 60	2	Single	3	18 and OVER	3
*Victor*	51 to 60	20	Single	3	Not Mentioned	3
*Raul*	More than 61	25	Married	5	Not Mentioned	10

Most participants were married (13/18), except for one widower, three single men, and one divorced. Only four of the participants held white-collar jobs (manager, lawyer, journalist, and community organizer); most (14/18) held blue collar jobs such as taxi or truck drivers (64%), carpenter, fisherman, electrician, artist, and unskilled worker. Overall, the type of work the interviewees reported translated into a variable (and even unreliable) monthly income. For instance, the income of a taxi, moto taxi, or truck driver depends on the number of passengers or truckloads you can transport per week or day. Furthermore, these occupations require a good physical condition and often makes it difficult to follow the diet recommended for people living with diabetes, as they rarely go home for their meals. Keeping up with the dietary restrictions prescribed by doctors was by far one of the biggest challenges for the participants. It was clear from their accounts that family members (especially their partners and daughters) were actively involved in assuring they followed the doctor’s dietary recommendations. Family members would provide support in preparing food for participants (ensuring meals were balanced – not eating outside food); reminding them to be careful with their diet and to avoid certain foods; and adapting their own diets to eat the same food as the participants in order to avoid the participant feeling unsupported or alienated. This last action was something the participants really appreciated, as they saw it was a way in which their family members expressed concern and love.

All participants were enrolled in the National Insurance Plan (*Seguro Integral de Salud*) and could receive free care for their condition at public health facilities and receive medications at no cost. However, some of them stated that when they had some extra money, or with the support of family members, they would visit private doctors and/or buy the medications in a private pharmacy to avoid the waiting times at public health facilities. They explained that getting an appointment at a time that was convenient for them was not always possible and given that the medications provided for free were not particularly expensive, they would oftentimes buy them out of pocket. It is important to acknowledge that, based on their accounts, it seems this inconsistent use of healthcare services sometimes translated into an increase in their glucose levels that “forced” them to go back to the health facilities to receive care.

Family support was a core component on their capacity to follow the doctor’s recommendations. Participants had multiple social roles within the family (they were fathers, sons, siblings, and spouses). However, we want to focus on the way these men weaved their responsibilities and commitment as fathers in their descriptions of their efforts to both maintain a healthy lifestyle and manage their diabetes. To ensure the anonymity of participants, we use pseudonyms throughout this manuscript.

### 4.2 A father must “be there” for his children

Being a father appears as a source of strength for complying with behavioral changes. The importance of fatherhood became evident as participants shared the challenges they faced to introduce behavioral changes into their everyday lives. Some of these changes included the need to keep up with taking their medications as indicated by their doctors, increasing their consumption of vegetables, and reducing their alcohol intake. Participants stressed the importance of following doctor’s orders so their health would not deteriorate, causing them to be absent in their children’s lives. One of the ways participants demonstrated their feelings of responsibility towards “being present” in the lives of their children was through expressions that highlighted the fear of dying and/or being too sick to spend time with their children and watch them grow up.

*“[I tell my daughters] ‘I will continue to live for you. I don't want to collapse, I don't want to die'; because that's what I don't want. I tell them: 'I want to continue living and spend with you the time that life gives me … I'm going to stay alive because I have to see you. You have to leave before me. I'm not going to leave.’” (Arturo, 34 years old, 4 children).*

Similarly, other participants stressed the importance of wanting to see their children grow up and to take care of them. This was identified by the participants as an important reason to take their diabetes care seriously:

*“I have young children, I mean, right? If I don’t control [my diabetes] to live longer, [if] I don’t control what I should eat and what I shouldn’t eat, I won’t see my children grow up. That has been a decisive point to change what I ate before from what I eat now.” (Mauro, 55 years old, 9 children).*

As stated above, being healthy in order to care for their children was also mentioned as a source of motivation for men:

*“Diabetes is a very painful disease if you don’t know how to take care of yourself. So, I took it that way [seriously] because my daughters are small (…) they are 4 and 6 years old. So, I took it very, very seriously the first two months because I felt that if I left, who was going to take care of them? As a child, I never met my father, and I did not want them to go through the same thing…” (Giacomo, 59 years old, 2 children).*

The possibility of abandoning their children as a consequence of dying was a source of worry for these participants who had small children. Giacomo shared that after a particularly critical health problem related to having high glucose levels, his wife left him and took their children. This event made him realize that he had not been taking care of his health and that in turn, he was not really taking care of his children, and being a good father. The participant’s worries are connected to both caring and providing for his children, which are important components of being a good father. In the case of the nine participants with adult children, the concern of not being present or being physically able to care for their children had dissipated:

*“Well, sometimes I don’t worry so much [about taking care of my diabetes], so to speak, right? I don’t have small children. If I had little ones, how would I be? My children are adults now … our children are all in a relationship, and one no longer lives for them….” (Samuel, 51 years old, 4 children).*

This participant acknowledges that if his children were young, he would worry more about his condition. However, those with adult children did express concern about their children developing diabetes and would advise them to take care of their health.

One interesting thing to point out is that at least two participants shared that their doctors also used their responsibility as fathers to encourage them to follow and maintain treatment. Both Dario and Helder remembered doctors saying that if they did not take care of their health, they will not live to see their families grow:

*“If you want to be with your family, you have to take care of yourself because this is serious; this is not a game.” (Dario, 57 years old, 2 children).*

*“Don’t you want to meet your grandchildren? because at that time I did not have grandchildren. Don’t you want to live longer? So, I became motivated and went on a diet.” (Helder, 58 years old, 5 children).*

In the case of Dario and Helder, who had adult children, it was their grandchildren (or the possibility of having grandchildren) that translated into a motivation for staying healthy. Several participants also mentioned the impact of diabetes in their capacity to work, and the consequent fragile employment status was a particular source of worry for these men, as it affected their ability to “take care” of their families and support them financially.

### 4.3 A father must be a good example for their children

Some participants used their duty as fathers to be a role model for their children as another reason to follow diabetes management recommendations. Sometimes, they highlighted that being a good role model was important to encourage children to follow their steps, as in many cases they mentioned that diabetes “runs in the family”. Others used their poor health to educate their children on the negative consequences of not being responsible with your lifestyle choices.

#### 4.3.1 Being strong and responsible

Some participants stated that being responsible in the management of their condition was an opportunity to show their children they are strong and that they do not give up in the face of adversity. Diabetes was seen as a condition with which they needed to fight. One participant shared what he said to his daughters when he broke the news of his diabetes diagnosis:

*“I’m going to take a treatment and maybe you’re going to be shocked [because] suddenly I’m going to lose a little more weight, and I’m going to keep fighting. My eyes filled with tears because my daughters looked at me and said, “Dad, are you going to die?” “No, I tell you. I’m not going to die because this isn’t going to kill me. What is going to kill me is your disagreements. That’s what’s going to make me die faster…. for my family I have to fight until the end, until God gives me life.” (Arturo, 34 years old, 4 children).*

Later, this same participant used the metaphor of a castle to show the role he played for his daughters and his family as a whole, and the reason he needed to stay strong despite having diabetes:

*“My family says: you are the head of the family. And if you fall, we are all going to fall, because you are the pyramid you forged. You are the castle, if the castle falls on the floor, the rest of us [are] all defeat[ed] because everyone is going to pass over us. It’s like cards, you’re the ace, and we follow behind you. If we don’t follow you, we are nobody. We are nobody, in this life we are nobody yet. You are the one who guides us, you are the person who, no matter how much bread you have, you know how to share.” (Arturo, 34 years old, 4 children).*

Another participant stated that the love of his family, but especially his children, gave him strength to follow the lifestyle changes needed to manage his diabetes:

*“They [his children] don’t abandon you. And that strengthens you. How am I not going to stop drinking if I see that they love me so much” (Raul, 68 years old, 5 children).*

Closely related with the idea of being strong for their families was the importance of being self-sufficient, and not increasing their family’s obligations towards them, as Giacomo stated:

*“The factors that motivate me to continuing taking care of myself, to health myself are to depend on myself, right? … another thing is the fear of being a burden to the family. So, I wouldn’t want to get a sick and depend on being taken care of, in that part I’m a little proud too. I want to depend on myself. (Giacomo, 59 years old, 2 children).*

In this case, we see how the importance of being emotionally strong and self-sufficient, which are common themes in masculine gender norms, intersect with their duties as fathers that ought to be about providing for your family rather than burdening them with your care.

Others were worried about making their loved ones suffer because of their condition. Jorge’s testimony shows his concern about both disappointing his daughters and making them worry about his health.

*“Sometimes they cry when they discover that I have eaten a cookie or had a glass of soda. And they’ve cried because I can’t eat [the same things they eat]. [When I was diagnosed with diabetes] my wife talked to them a lot about the disease and they know that if I don’t take care of myself, I’m going to die in a very ugly way, let’s say, in a very sad way. So, they sometimes cry when they see me do that [not follow dietary restrictions]and I have to quit. … I’m taking other herbs for [diabetes] that have helped me a lot [because] I do not want to end my life causing others to grief for me or being a burden to the family but being able to help my daughters. Be strong until they are grown-ups.” (Jorge, 39 years old, 2 children).*

Jorge is particularly sensitive to the fact his young daughters (aged 4 and 6) are constantly worried about his health and about the possibility of him dying, which is influenced by the way the mother informed them of his diagnosis. Jorge feels an obligation to reciprocate the concern his daughters have for him, and he expressed feelings of guilt when he failed to follow the dietary guidelines prescribed by his doctor.

#### 4.3.2 Not giving up

Participants also talked about another important way of being an example for their children by demonstrating their capacity to keep the progression of the disease at bay. The importance of being strong is not only to “fight” the disease but also a lesson for their children on how to face challenging situations:

*“I don’t want that. I want to continue living, I want to continue showing them that I can face this, that even if I stumble and fall, I will continue to get up and I will continue walking. [I tell them]: ‘You are going to see me until I am old, you are going to come to change my diaper because I will not be able to change it myself.’ ‘Oh, Dad, don’t exaggerate!’” (Arturo, 34 years old, 4 children).*

Finally, there were several instances in which along with their concern about being a good father, came the concern of being a good son, one that does not create worries for his mother or additional expenses and demands. Thus, their worry about not causing any distress in his loved ones expanded beyond their children and included their mothers and spouses:

*“Now, what motivates me [to take care of my diabetes] are my children. Now that I have my grandson who is just 4 months old, those are the people who motivate me. My wife, my mom too. More than anything [I take care of myself] for my mom because she is the one who suffers the most when she sees her son like this. I know my mom suffered when she found out I had diabetes*.” *(Facundo, 54 years old, 2 children).*

Other participants delayed telling their mothers about their condition, particularly those whose fathers also had diabetes or had died because of improper management of their condition. Although the responsibilities associated with fatherhood and being a son are very different, one commonality is the importance of avoiding being a source of worry or suffering to your family, be it your children, parents, or partner.

### 4.4 The changing roles as fathers: the relevance of temporality

As we examined our data, it became evident that the age of their children (and thus the weight of their responsibility towards them) played an essential role in their attitudes and actions towards the behavioral changes they had to incorporate in their lives since their official diagnosis. It was clear that men with younger children saw them as a key motivator to stay healthy, either because they did not want to miss-out on their lives by passing away prematurely or by not being able to play and spend quality time with them because of their deteriorating health. Interestingly, the act of dying when their children were still young was presented by participants as abandoning their children, which shows the responsibility they feel towards being active in their children’s lives and providing for them, both in the present and future.

It became apparent that fatherhood (like other social roles) undergoes changes over the life cycle. As children grow and become adults, the duties and responsibilities of parents change, oftentimes are reduced, or at least undergo a transformation. In this sense, the concept of temporality applied to the roles and responsibilities associated to fatherhood is particularly relevant to understand the fluidity of men’s illness experiences with diabetes and motivation to incorporate and sustain the wide diversity of actions necessary to maintain healthy glucose levels.

## Discussion

Through an analysis of men’s accounts regarding their efforts to stay healthy amidst a diabetes diagnosis, we show that being a father plays a central role in their motivation to stay healthy. Our study contributes to studies aimed at analyzing the relevance of masculine gender norms to understand both health promoting behaviors and detrimental ones. Masculinity studies have looked at the way men are socialized, how men’s roles are socially constructed, and how these roles change over the life cycle and in different social contexts. Previous studies suggest the social construction of masculinity can produce social norms that either positively or negatively reinforce the adoption of health compromising behaviors (5). However, very few studies in Latin America use this approach to understand men’s health-related behaviors beyond sexual and reproductive health (20). Our study contributes to understanding the elements of masculinity that could provide insights to design gender and context appropriate health promotion interventions for men with diabetes.

The participants’ accounts included several instances where a variety of family members encouraged them and supported them in their efforts to stay healthy. However, being a father, and the rules and responsibilities attached to such a role, constituted a key motivation for disease management. The men’s perceptions of their obligations and responsibilities associated with fatherhood were a source of strength for staying healthy, and a source of worry when they felt their health was deteriorating. Based on the participants’ accounts, we understood that parenting implies being physically present in the lives of their children as they grow, rather than being a burden of them due to sickness or premature death. Participants also feel obliged to reciprocate the concern and actions demonstrated by the children – such as, preparing special food for them, reminding them to take their medications, and to avoid certain foods and drinks.

Our paper advances current studies around the intersection of masculinities and health. We are aware that there are multiple ways of being socially recognized as men, with a diversity of co-existing versions of what it means to be a man in the same setting (27). Our study contributes with studies aimed at understanding how masculinity influences health behavior among men living with diabetes. In this article, we showed that fatherhood constitutes an important element of masculine identity and that their roles as fathers constitutes both a motivation to comply with diabetes management regimes, and as a source of worry and concern if they are unable to do so. Research exploring how masculinity influences key health outcomes among men living with diabetes in LMIC through biological, affective/emotional, and behavioral pathways is long overdue. In this sense, we echo, some of Jack et al’s (5) ideas around the components that need to be understood to design and identify strategies tailored to men with diabetes, such as the influence of masculinity in health-decision making, psychological well-being, quality of life, and health care– seeking behaviors.

Our study points to the relevance of understanding expectations around fatherhood in the illness experiences of men. Illness experiences are not homogeneous across individuals as they reflect a person’s subjective experience with a particular disease (14). Illness experiences vary across individuals who suffer from the same condition and across the lifetime of an individual affected by a chronic condition, as both biological and social factors play a role in the changing meaning and perception of illness experience (14, 15). Our research shows the relevance of taking into account the changing social expectations towards individuals as they assume new social roles (fathers) and age.

We also contribute with a more nuanced way of thinking about the impact of living with a chronic condition in a person’s biography, looking not only at the disruptive impacts but also the opportunities for resilience that may arise from the need to reconfigure one’s sense of self-worth. Bury (8) has called attention to the fact that chronic conditions undermine the ability of individuals to perform certain actions in their everyday lives and fulfill their responsibilities and roles, affecting their social status. Charmaz (9) has also highlighted the negative implications of chronic conditions in the self-image of a person and in its capacity to develop an equally valuable alternative of self. We agree with these authors that living with a chronic condition constitutes a biographical disruption, but our research provides evidence about the efforts men undertake to ensure “biographical continuity” (28). Understanding those aspects of men’s life that enables such continuity, such as being a caring and supportive father, could provide insights about the components that ought to be part of any effort to promote healthy behaviors among men. As Jack et al. (5) state, it is important to listen to the way the individuals affected by a chronic condition find meaning and strength in order to design gender-appropriate diabetes management interventions. Our study advances the research around the intersection between health and gender norms and argues that a more nuanced understanding of the construction of masculinity and the relevance of fatherhood in the lives of men could be useful to design and identify better health promotion strategies tailored to men with diabetes.

Our study has the following limitations; on the one hand, since it was not originally focused on fatherhood, we did not explore the meaning of such role directly with participants. Rather, we deducted it from their narratives. A new study interested in this issue should include this topic in the conceptualization of the research and ensure it is present throughout the data collection and analysis. Additionally, because the population was only Peruvian men, we cannot speak for all Latin-American men and their experiences, or of all Peruvian men such as those who live in rural areas or who belong to other socioeconomic strata who do not use public health services.

A future study aiming at understanding the influence of the role of temporality in men’s experiences with chronic condition should have a longitudinal design, to see if the ages of their children (as they become less dependent on their parents) play a role in the importance of fatherhood as a motivator for staying healthy.

## Data availability statement

The data that support the findings of this study are available on request from the corresponding author (MAP). The data are not publicly available due to transcripts containing information that could compromise the privacy of research participants.

## Ethics statement

The studies involving humans were approved by Dickinson College Institutional Review Board. The studies were conducted in accordance with the local legislation and institutional requirements. The participants provided their written informed consent to participate in this study.

## Author contributions

MP designed the original study, the data collection tools and was responsible for the data collection process. MP and IF analyzed and interpreted the data with support from JM. MP and IF wrote the first draft of the manuscript and received critical and thorough feedback from JM. All authors contributed to the article and approved the submitted version.
